# Trans Adults Amidst the COVID-19 Pandemic: Quality of Life, Pandemic Impact, and Vaccine Preferences

**DOI:** 10.3390/ijerph182312536

**Published:** 2021-11-28

**Authors:** Vern Harner, Ascher K. Munion, Jama Shelton

**Affiliations:** 1 School of Social Work, University of Washington, Seattle, WA 98105, USA; 2 Department of Psychology, East Carolina University, Greenville, NC 27858, USA; muniona20@ecu.edu; 3 Silberman School of Social Work, City University of New York, New York, NY 10065, USA; jshelton@hunter.cuny.edu

**Keywords:** transgender, nonbinary, COVID-19, quality of life, vaccines

## Abstract

The ongoing COVID-19 pandemic is disproportionately impacting marginalized communities, such as Black, Indigenous, and people of color (BIPOC), disabled individuals, and transgender/nonbinary (i.e., trans) individuals. As trans individuals may be multiply marginalized, it is necessary to examine within group differences among trans individuals of different genders, races, socioeconomic statuses, and abilities. This study examines the following research questions: (1) What is the quality of life of trans adults during the COVID-19 pandemic? (2) How does the self-reported impact of the pandemic vary across groups within the trans community? (3) What preferences do trans adults have regarding receiving a COVID-19 vaccine? Survey data were collected in August/September of 2020. Among a sample of 449 trans adults, findings suggest that the profound impact of the pandemic was not consistent across all community members. Being a woman predicted a higher self-reported impact of the pandemic while being a masc(uline) white respondent tended to predict a lower impact of the pandemic. Higher income was associated with a higher quality of life and being a disabled white respondent predicted a lower quality of life. The majority (99%) of the sample reported wanting to receive a COVID-19 vaccine should one become available. Implications for practice include the importance of considering the holistic experiences of clients and community members, as opposed to having homogenized perspectives of even subsets of the trans community. Future research related to barriers faced when attempting to access a vaccine is needed to inform future public health responses to epidemics/pandemics impacting this community.

## 1. Introduction

The ongoing COVID-19 pandemic is disproportionately impacting marginalized communities, such as Black, Indigenous, and people of color (BIPOC), disabled individuals, and transgender/nonbinary (i.e., trans) individuals [1,2,3]. Within the trans community, individuals may be multiply marginalized—that is, facing systemic oppression from more than one axis at once. For this reason, it is necessary to examine within group differences among trans individuals of different genders, races, socioeconomic statuses, and abilities. With this in mind, exploring differences within the trans community in regard to quality of life is a useful lens, especially during stressful societal events with differential impacts, such as the pandemic. The World Health Organization (WHO) defines quality of life as an “individuals’ perceptions of their position in life in the context of the culture and value systems in which they live and in relation to their goals, expectations, standards and concerns”, which includes the domains of physical health, psychological, social relationships, and the environment [4]. Studies of specific marginalized populations (e.g., people of color, older adults, disabled) have shown the connection between these domains, illustrating community-specific impacts between social relationships and health (e.g., lower mortality and higher self-reported health among older adults and African Americans) [5,6].

Disparate outcomes in transgender physical and mental health are increasingly well documented by researchers. Trans populations have been shown to have multiple increased risk factors (i.e., lack of acceptance, mental illness), a lack of protective factors (i.e., accepting families, a sense of community), and unmet needs (i.e., culturally competent medical and social service provision) [7,8,9,10,11,12,13]. Notably, the 2015 U.S. Trans Study (*n* = 27,715) found that 40% of transgender adults reported having attempted suicide at least once [11].

Despite documented increased risk factors, including heightened risk for HIV, HPV, and other conditions [14,15,16], trans individuals may avoid seeking medical attention. Nearly a quarter of respondents in the U.S. Trans Survey reported not seeing a doctor when they needed to for fear of being mistreated [11]. While a growing body of literature examines the experiences of trans individuals and healthcare providers, relatively little is known regarding attitudes towards or access to vaccines in trans communities. Multiple studies have found trans individuals partake in vaccine research to do their part to protect their community [14,15]. Andrasik and colleagues [14] found that trans individuals (*n* = 13) were more likely to maintain low HIV risk profiles and follow trial procedures than their cisgender (cis) counterparts (*n =* 681). In one study (*n* = 3529), Rutherford and colleagues [16] found that, while more likely to report being vaccinated for HPV than their cis counterparts, trans respondents were also more likely to report being denied the HPV vaccination than sexual minority cis men in the sample (4.1% vs. 1.6%). In their qualitative examination assessing knowledge of HIV vaccines among trans women, Taylor et al. [15] also identified barriers to HIV vaccine access, including fear of side effects or feeling excluded from biomedical research.

Trans individuals may also have lower access to or lack of trust in social services and healthcare due to lack of health insurance, poverty, a history of negative experiences, and/or lack of competent providers [17,18,19]. This lack of access is compounded during the COVID-19 crisis, as gender-affirming medical treatments were often postponed due to healthcare systems having to limit capacity [20]. As receiving necessary gender-affirming surgeries has been shown to be associated with redacted mental health problems, this is a dual crisis pertaining to both the physical and mental wellbeing of trans individuals [21]. Continued hormone replacement therapy is also a concern during the pandemic, as medical support and hormone access may wane [22]. Despite difficulties in accessing healthcare during the pandemic, there is some evidence that, through the availability of telehealth appointments, some trans individuals were able to initiate or continue their gender-affirming care in 2020 [23].

The COVID-19 pandemic provides a unique environment with unknown consequences for our mental and physical wellbeing. Social relationships (which may be impacted by social distancing) have been shown to have a causal association with positive health outcomes [24,25,26]. This research has shown the impact of social networks and support on both physical and mental health. High levels of social capital, that trans individuals might lack, have been linked to lower mortality rates and positive perceptions of health [27,28]. During a crisis like COVID-19, social support, coping, and resilience play an integral role in buffering the extreme levels of stress and anxiety faced by both the general population and marginalized communities such as trans, BIPOC, and/or disabled [29,30]. Umucu and Lee [31] found that, in a sample of individuals with disabilities and/or chronic conditions (*n* = 269), perceived stress related to COVID-19 was positively associated with both adaptive and maladaptive coping strategies such as self-distraction, denial, substance use, behavioral disengagement, venting, planning, religion, and self-blame.

Additionally, multiple studies have identified the predictive nature of self-perceived general health of chronic disease, use of medical services, disability, and mortality [5,6,32,33]. In a sample of 477 LGBTQ college students aged 19–25, Gonzales et al. [34] found that 79.1% of trans respondents reported experiencing frequent mental distress during April–June 2020. Comparatively, 36.5% of cisgender LGB men and 58.4% of cisgender LGB women reported experiencing frequent mental distress in the same time period. This study also found that nearly half (45.7%) of the sample reporting their immediate families—who they may or may not be quarantining with, did either not support or know about their LGBTQ identity. Studies of specific vulnerable populations (e.g., people of color, older adults, disabled) have shown community-specific impacts of the connection between social relationships and health [5,6]. The lack of social connection due to quarantining or isolating during the COVID-19 pandemic has a unique impact on trans and queer populations, including youth, who may lose a sense of a queer/trans community as a result [35]. This impact may be even more exacerbated on immigrant, Latine, and broader BIPOC communities within the trans community who are especially targeted by anti-trans violence [36]. Although there are similarities between the ways cisgender sexual minorities (i.e., LGB) navigate mental/physical health, trans communities have experiences unique to them and, therefore, it is important for researchers to examine these issues through a trans-specific lens with a focus on within group differences [22,37].

This is the first pandemic of this magnitude faced by the trans community since the 1980s, which was prior to the social sciences recognizing and studying the trans community separately from the broader LGBTQ community. To that end, it is necessary for social science scholars to consider the experiences of trans individuals in the face of a global health crisis. The current study contributes to the sparse existing knowledge of the wellbeing of trans individuals during the COVID-19 pandemic by addressing the following research questions: (1) What is the quality of life of trans adults during the COVID-19 pandemic? (2) How does the self-reported impact of the pandemic vary across groups (e.g., race, gender, class, ability) within the trans community? (3) What preferences do trans adults have regarding receiving a COVID-19 vaccine?

## 2. Methods

This cross-sectional survey was collected in August and September of 2020. Study data were collected and managed using REDCap electronic data capture tools hosted at the University of Washington [38,39]. This study (STUDY00010863) was approved by the University of Washington Institutional Review Board on 4 August 2020 and respondents completed an informed consent.

### 2.1. Sample and Recruitment

Participant include adults (18+) who are transgender, nonbinary, and/or some other gender minority or who have a history of transitioning their gender. Respondents were also required to read/write in English and access the survey online. Using g* power, with alpha set at 0.05, power (1-beta) set at 0.80, and assuming a small effect size (f^2 = 0.1), a sample size of 81 determined to be necessary to detect significance on the least-powered parameter in the intended analyses (betas in the multiple regression). Recruitment was conducted via social media (i.e., Facebook groups, ads on Instagram/Facebook) and professional/community networks (i.e., LGBTQ Caucus of Faculty & Students in Social Work listserv). Respondents were able to enter a raffle for a chance of one of five $25 gift cards.

### 2.2. Measures

To address the research questions related to COVID-19, 55 variables of the larger dataset of 256 variables are included. Additional QOL, community belonging, and social support data not related to COVID-19 and constituting the additional 200 variables—collected to assess other hypotheses—are not examined in these analyses. The current study includes 26 demographic variables such as age, gender, race, relationship status, political identity, gender expression, and disability status. Demographic variables were informed by the Behavioral Risk Factor Surveillance System (BRFSS) [40] and the U.S. Trans Survey [11]. Additionally, items related directly to the COVID-19 pandemic and respondent QOL were included and are detailed below.

#### 2.2.1. BRFSS

Utilizing 13 items from the BRFSS [40], respondents were asked about their health and wellbeing, access to services, and social determinants of health. This included disability status, access to health insurance, and satisfaction with the healthcare they have received in the past year.

#### 2.2.2. WHOQOL

Utilizing a selection of Likert scale items from the WHOQOL-BREF [4] respondents were asked questions pertaining to the four domains of QOL, as defined by the WHO (i.e., physical health, psychological, social relationships, and environment). For example, respondents were asked how safe they feel in their daily life, how often they had negative feelings in the past two weeks, and how satisfied they were with the quality of their sleep and sex life in the past two weeks. 

#### 2.2.3. COVID-19

Respondents were asked about the impact of COVID-19 on their lives and their preferences around a vaccine (i.e., if/when/how they would want to receive it). This also included items asking how many days they had cared for someone or worked outside their home in the past month.

### 2.3. Analysis

Proportions of missing data ranged from 2.67% (*n =* 12) (sexual orientation) to 22.27% (*n =* 100) (number of times visited a health professional in past year). Missing data was handled via generation of 50 imputation files using the mice package’s predictive mean matching in R [41,42]. Descriptive statistics, visual inspection of the data, and iteration histories were examined to ensure convergence. Relative efficiency was above 0.95 for all analyses indicating effective imputation. All analyses excluding descriptive statistics were performed on the imputed sample using SPSS 28 [43].

## 3. Results

### 3.1. Describing the Sample

Respondents include 449 individuals from 38 states (plus Washington D.C.), (*n =* 371) and 12 countries (including the United States). While 45 respondents indicated they lived in a country other than the United States, 27 of these were from Canada. Other countries included in the study (i.e., England, Norway, Korea, Argentina, Australia, Germany, Ireland, Scotland, Spain) were only represented by 1 to 7 respondents each. While all respondents were trans (including binary, nonbinary, and culturally specific identities), when asked to indicate which simplified gender category they most identified with, 279 (62.14%) respondents indicated they most identified as nonbinary, 114 (25.39%) as men, 51 (11.36%) as women, and 5 (1.11%) as a culturally specific gender. The sample was majority white (*n =* 335, 74.61%) or bi/multiracial (*n =* 64, 14.25%) (Table 1).

The mean age was 31.69 years and 65.92% (*n =* 296) were disabled and/or chronically ill. While 77.28% (*n =* 347) had completed an associates, bachelors, masters, or doctorate degree, 37.19% (*n =* 167) earned less than $20,000/year and 65.70% (*n =* 295) earned less than $40,000/year in 2019. Age was moderately correlated to income (r(447) = 0.29, *p* < 0.001), with the average age of those earning $75,000/year or more was 37.65 years, while the average age of those earning under $25,000/year was 29.51 years. When asked about their activity over the last month, 7% reported caring for someone else and 23% reported having worked outside their home for 14 or more days. Nine percent (*n =* 42) reported being unemployed due to the pandemic and 10% (*n =* 47) reported being unemployed due to other reasons. While the majority of the sample (*n =* 371, 82.63%) had begun transitioning (i.e., socially, legally, and/or medically aligning their gender) or had reached all of their transition goals, about two thirds of the sample (*n =* 296, 65.92%) were out to most people, almost everyone, or everyone in their lives. Additional demographic information of the sample is presented in Table A1 in Appendix A.

A quality of life score was computed using eight items from the WHOQOL-BREF measure, with a maximum score of 37 possible. Respondent’s QOL scores ranged from 15–31 (sd = 2.944, with a mean of 23.21 (Table 2)).

### 3.2. Predicting COVID-19 Impact & Quality of Life

Quality of life and self-reported impact of COVID-19 were found to be negatively correlated (r(447) = −0.111, *p* = 0.046), indicating an association between higher quality of life and lower impact of the pandemic. Quality of life was also positively correlated with annual individual income (r(447) = 0.123, *p* = 0.018). Additionally, being disabled and/or chronically ill was negatively correlated with quality of life (r(447) = −0.248, *p* < 0.001) and positively correlated with self-reported impact of the pandemic (r(447) = 0.116, *p* = 0.044).

Multiple linear regression models predicting the self-reported impact of COVID-19 were created. All efficiencies were reported at 0.95 or above. First, the variables of age, individual income (in U.S. dollars), gender, and race (0 = white, 1 = BIPOC) were tested as predictors. In the model predicting COVID-19 impact (R2 = 0.024, F(1449) = 0.272, *p* = 0.047), only being a woman significantly predicted a higher self-reported impact of the pandemic (Table 3).

To compare predictors of QOL and the impact of the pandemic, the variables of age, individual income (in U.S, dollars), gender, and race were tested as predictors of QOL. In the model predicting QOL (R2 = 0.033, F(1449) = 0.085, *p* = 0.006), only income significantly predicted higher QOL (Table 4).

Next, the variables of QOL, being disabled/chronically ill, fem(me), and the degree to which the respondent was out as trans to others were tested as predictors of the self-reported impact of the pandemic. In the model predicting COVID-19 impact (R2 = 0.024, F(1449) = 2.736, *p* = 0.081), no predictors reached statistical significance (Table 5).

### 3.3. Results by Race, Ability, and Gender

Gender identities (i.e., man, woman, nonbinary) and gender expressions (i.e., whether someone was masc(uline), fem(me), or androgynous) were found to be correlated with one another (Table 6). Gender expression categories were not mutually exclusive, as respondents may have fluid or multi-faceted ways of presenting themselves. Both odds ratios using a logistic regression and linear regression were used to examine intersectional experiences based on race, ability, and gender expression. While highly correlated, we utilized gender expression in subsequent analyses, since it is likely more consistent with social perceptions of individuals’ genders and may, therefore, have larger influence on how individuals are treated by others (as compared to internal gender identity).

Dummy variables for fem(me) BIPOC (*n =* 29, 6.4%), masc(uline) white (*n =* 172, 38.3%), masc(uline) BIPOC (*n =* 53, 11.8%), fem(me) white (*n =* 97, 21.6%), white and disabled (*n =* 216, 48.0%), BIPOC and disabled (*n =* 76, 17%), BIPOC, nondisabled white (*n =* 118, 26.28%), and nondisabled BIPOC (*n =* 34, 7.57%) respondents in the sample were created so that the relationship between these groups and independent variables of interest could be examined (Table 7). Though some groups had slightly higher or lower odds of being low income, they failed to reach significance. While masc(uline) BIPOC respondents had 56% higher odds (*p* = 0.043), disabled white and disabled BIPOC respondents had lower odds (45% (*p* < 0.001) and 27% (*p* = 0.009), respectively) of reporting feeling “very much” or “extremely safe” in their daily life during the past two weeks.

Next, QOL and COVID-19 impact were predicted using linear regression based on the above intersecting identity groups. In this model (R2 = 0.074, F(1449) = 5.893, *p* < 0.001), disabled white respondents reported lower qualities of life (*p* < 0.001) than other groups (Table 8). In the model predicting COVID-19 impact (R2 = 0.043, F(1449) = 3.315, *p* = 0.014), being a masculine white respondent predicted reporting a lower impact of the pandemic (*p* = 0.025) (Table 9).

### 3.4. Vaccine Preferences

Utilizing non-mutually exclusive items, respondents were asked about their preferences regarding receiving a COVID-19 vaccine (Table 10). Overall, 98.9% (*n =* 444) of the sample (99.6% of white respondents and 96.7% of BIPOC respondents) reported they would want to receive a vaccine should it become available. While just over a third of respondents (36.5%, *n =* 164) indicated they would want to receive a COVID-19 vaccine as soon as possible (37.9% of white respondents and 32.4% of BIPOC respondents), a third of respondents (*n* = 150, 33.4%) reported they would only want to receive it after side effects had been shown to be minimal or manageable. Additionally, a third of respondents (33.0%, *n =* 148) reported they would only want to receive the vaccine after other, more vulnerable groups (such as chronically ill individuals or older adults) had been able to receive it. While 12.7% of respondents (*n =* 57) would prefer to wait until it had been used by others for at least one month, 9.1% (*n =* 41) and 2.2% (*n =* 10) of the sample would prefer to wait 6 months or a year, respectively. Only 0.4% of the sample (*n =* 2) would only want to receive it if available via nasal spray instead of a shot.

Almost half (45.99%, *n =* 63) of individuals with both mental and physical health conditions would prefer to receive the vaccine as soon as possible, while only 12.3% (*n =* 14) of individuals with neither physical nor mental health conditions would opt to do so. Additionally, individuals with both physical and mental health conditions (*n =* 56, 40.9%) reported preferring to wait until the vaccine had been shown to have minimal side effects (as compared to 7.9%, *n =* 9 of those without health conditions).

While almost half (*n =* 23, 45.1%) of respondents who earned $75,000/year would prefer to receive a vaccine as soon as possible, only 25.5% (*n* = 13) would wait until other, more vulnerable groups had received it. Almost half (*n =* 85, 45.5%) of individuals with only mental health conditions (with no physical health conditions) reported preferring to wait until other, more vulnerable groups had received a vaccine, though a similar number of these individuals reported wanting to receive the vaccine ASAP (*n =* 84, 44.9%) or after minimal side effects had been shown (*n =* 78, 41.7%). Roughly a third of the BIPOC in the sample (*n =* 84, 44.9%) reported wanting to be vaccinated as soon as possible (*n =* 36, 32.4%), while 30.6% (*n =* 34) would want to wait until minimal side effects had been shown.

## 4. Discussion

This study sought to examine the quality of life and vaccine preferences of trans adults during a snapshot of a global pandemic. Given the dearth of literature on vaccine preferences among trans individuals, this study makes an important contribution to the existing literature. Collected during August and September of 2020, these findings suggest that, while the COVID-19 pandemic profoundly impacted the trans community, this impact was not consistent across all community members. As reflected in many other studies, trans individuals of different identities do not move through the world as one identity at a time [9,11,22,34,37]. For this reason, analytic methods to consider multiple axes of identity/experience at once were used. In addition to informing provider-client interactions, findings from this study can be used to inform vaccine booster roll-out, programs in response to the COVID-19 pandemic or other crises, and health-related policy.

After COVID cases reached a second peak in the U.S. in mid-July, they slowly decreased from July to mid-September (when they began increasing once more) [44]. On 31 August 2020, the 7-day average number of new cases in the U.S. was 41,6000. However, the geographic region where respondents were residing at the time of data collection had a variety of policies and practices in place in response to the pandemic. The states with the most respondents included: Washington (*n* = 122), Illinois (*n* = 35), and California (*n* = 22). In California, COVID-19 positivity rates were hitting a record low in September, following outbreaks in state prisons and implementing the “Blueprint for a Safer Economy framework” the month before [45,46]. Using the Blueprint framework, each county in California was assigned a risk-level tier based on positivity rate, adjusted case rate, and/or health equity metric [46,47]. Though rates were lower than in July and August of 2020, as of 31 September 2020, 38 of the 58 counties were in Tier 1 [46,47]. This tier indicated the virus was widespread and meant most schools were closed to in-person instruction, there were more than 10 daily new cases per 100,000 residents, and indoor business operations were suspended or severely limited [46]. Meanwhile in Canada, cases very slightly peaked in July 2020 (after easing some restriction to in-person activities early in July) and then increased steadily from early August 2020 through January 2021 [48,49]. In August and September, education and other in-person activities mostly took place with mask-wearing and social distancing [49]. As of 1 August 2020, there were 337 new COVID cases, compared to 2168 new cases on 1 October 2020 [48,49]. Thus, Canadian and international respondents, while still experiencing disruptions to daily life, had lower COVID exposure risk, yet were experiencing higher restrictions than US respondents.

While some research regarding the COVID-19 pandemic and quality of life is starting to be published, results regarding the specific impact on trans communities are still rare. One study regarding the impact of the first three months of the COVID-19 pandemic on trans Australians (N = 1019) [3] found that 49% of respondents reported thoughts of self-harm or suicide and 61.5% experienced clinically significant symptoms of depression. Further, half their sample reported experiencing financial strain and almost a quarter (22.4%) were unemployed [3]. More research is needed to better understand, and thus better serve, trans individuals during the current and future health crises.

COVID-19′s impact on cisgender community members is better understood. Mendes and Pereira [50] for example, found that LGB individuals (especially bisexuals) suffered more severe impacts of COVID-19 and had lower work-related QOL than heterosexual respondents, but did not examine the difference between respondents of different genders and did not ask respondents if they were transgender. In the current study, respondents were asked what the impact of COVID-19 had been on their lives overall (ranging from insignificant to catastrophic). Women overall in the sample reported a greater impact of the pandemic, though being masc(uline) and white or a nondisabled BIPOC respondent predicted reporting a lower impact of the pandemic. On average, disabled white respondents reported a lower QOL. Those with higher incomes reported a higher QOL, compared to those with lower incomes in the sample. Having a higher quality of life or being a masc(uline) white respondent was associated with reporting a lower impact of the pandemic. These findings align with other studies [51,52,53,54] that illustrate how varied experiences can be, even within subgroups (e.g., BIPOC, lower income, disabled) of larger communities.

Results from this study illustrate the profound differences of experiences within the trans community, especially in regard to disability. While between 15–25% of adults in the United States is disabled [55,56] about two thirds of the current sample reported being disabled and/or chronically ill. This is higher than the 39% of respondents in the U.S. Trans Survey who reported having a disability [11] or the 55% of the respondents in the Trans PULSE Canada study who reported being disabled or chronically ill [57]. In the current study, being disabled and/or chronically ill was associated with lower quality of life and higher self-reported impact of the pandemic. Though being disabled/chronically ill alone did not predict pandemic impact, being a white disabled and/or chronically ill respondent predicted reporting lower QOLs than other respondents, which aligns with prior research regarding disability and QOL [52,54]. Future analysis of the current study will more deeply examine the quality of life of disabled trans adults during the pandemic.

At the time of the data collection, no COVID-19 vaccine was publicly available yet. Distrust of the medical community, including vaccine provision, exists in multiple marginalized communities, including the trans community, due to historical and current mistreatment [18,58,59]. However, Matijczak and colleagues [60] found that gender and sexual minority (GSM) respondents were not more likely to delay or avoid testing/treatment for COVID-19, as compared to their non-GSM counterparts. Rutherford and colleagues [16] found that trans participants (*n =* 446) were more likely to report both being vaccinated for HPV and being denied the HPV vaccination than the sexual minority cis men (*n =* 3083) in the sample. Future research should examine whether trans individuals experienced barriers to receiving COVID-19 vaccinations and policy/practice shifts ensuring these barriers are not reproduced in the future must be made.

Findings from this study add to the burgeoning body of knowledge regarding differential experiences of trans individuals across multiple axes of identity and experience. The need to underscore the importance of emphasizing and honoring differences within communities remains. The variation in how the COVID-19 pandemic impacted different subgroups within the trans community, as well as the differences reported in quality of life, is no accident. Systems of oppression (including white supremacy, ableism, and misogyny) are utilized by individuals and institutions in power to heighten the impact of illness, poverty, and environmental issues on minoritized communities. When ensuring programs and practices are responsive to the needs of trans individuals, practitioners must not assume that an individual is either trans or BIPOC or disabled; rather, social service and healthcare provision should be shaped with the assumption that our clients hold multiple targeted identities. As these are systemic- and societal-level mechanisms, educators and practitioners must also include a focus on mezzo/macro interventions (e.g., policy, funding, culture shift).

### Strengths and Limitations

This study has multiple strengths and limitations that should be considered when interpreting findings. While disabled and/or chronically ill members of the trans community are highly represented within the sample, there are small subgroups (i.e., Alaskan Native, Middle Eastern, Pacific Islander) within BIPOC respondents. Additionally, though there were many genders represented in the sample, only 11.3% (*n =* 50) of the respondents identified primarily as binary trans women. However, as the data were collected in August and September of 2020, they provide a valuable snapshot of the experiences of trans individuals during the COVID-19 pandemic. While the sample is not a representative random sample, the sample does reflect a variety of regions in the United States and includes a subsample of individuals from outside the U.S. context. With 449 respondents, the sample size did allow for multivariate analysis and considering the experiences of groups by multiple facets of their identity/experiences (e.g., masc(uline) BIPOC).

## 5. Conclusions

Limitations notwithstanding, this study provides much-needed data about trans adults during the COVID-19 pandemic. Healthcare providers, social workers, researchers, and those involved in writing policy need data examining the experiences and needs of the trans community through multiple layered lenses—not gender modality alone—in order to ensure we are truly responding to the community’s needs and experiences. Future research related to barriers faced when attempting to access a vaccine, the experiences of disabled trans adults, and to better understand the impact of health crises on trans communities is needed to inform future public health responses to epidemics/pandemics impacting this community.

## Figures and Tables

**Table 1:** Respondent racial/ethnic identity.

Race/Ethnicity	*n*	%
Alaska Native	1	0.2%
Asian	13	2.9%
Black	7	1.6%
Latine	7	1.6%
MiddleEastern	1	0.2%
NativeAmerican	2	0.4%
PacificIslander	1	0.2%
White	332	73.9%
NotListed	1	0.2%
Decline	3	0.7%
Bi/Multi	65	14.5%
Missing	16	3.6%
Total	449	100.0%

**Table 2:** QOL descriptive statistics.

Variable	Mean QOL	Min	Max	Range	SD
Total Sample	23.21	15	31	16	2.944
Women	22.88	17	30	13	3.275
Men	23.46	16	31	15	2.748
Nonbinary	23.18	15	29	14	2.948
White	23.13	16	30	14	2.861
BIPOC	23.50	15	31	16	3.137
Income <25 k	22.92	15	30	15	3.097
Income between 25–40 k	22.99	15	31	16	3.210
Income between 40–75 k	23.72	17	31	14	2.560
Income >75 k	23.74	18	31	13	2.727
Disabled	22.67	15	31	16	2.983

**Table 3:** Predicting COVID-19 impact by demographics.

Predictor	b	SE	95% CI	t	*p*
(constant)	3.292	0.179	2.94, 3.64	18.405	<0.001 ***
Age	0.003	0.005	−0.01, 0.01	0.558	0.577
Income	−0.011	0.008	−0.03, 0.01	−1.333	0.182
Women	0.272	0.137	0.00, 0.54	1.984	0.047 *
Nonbinary	0.170	0.090	−0.01, 0.35	1.886	0.059
BIPOC	−0.128	0.089	−0.30, 0.05	−1.443	0.149

Note: Gender was represented as two dummy variables with men serving as the reference group. * significant at the *p* < 0.05 level. *** significant at the *p* < 0.001 level.

**Table 4:** Predicting QOL by demographics.

Predictor	b	SE	95% CI	t	*p*
(constant)	23.555	0.689	22.2, 24.91	34.171	<0.001 ***
Age	−0.030	0.017	−0.06, 0	−1.761	0.078
Income	0.085	0.031	0.03, 0.15	2.769	0.006 **
Women	−0.645	0.551	−1.73, 0.44	−1.170	0.242
Nonbinary	−0.359	0.348	−1.04, 0.32	−1.033	0.302
BIPOC	0.414	0.351	−0.28, 1.1	1.178	0.239

Note: Gender was represented as two dummy variables with men serving as the reference group. ** Significant at the *p* < 0.01 level. *** Significant at the *p* < 0.001 level.

**Table 5:** Predicting COVID-19 impact by QOL and biosocial factors.

Predictor	b	SE	95% CI	t	*p*
(constant)	3.809	0.384	3.05, 4.56	9.918	<0.001 ***
QOL	−0.022	0.015	−0.05, 0.01	−1.466	0.143
Disabled	0.143	0.096	−0.05, 0.33	1.491	0.137
Fem(me)	0.068	0.085	−0.10, 0.24	0.802	0.423
Degree of being out as trans to others	−0.009	0.024	−0.06, 0.04	-0.390	0.696

*** Significant at the *p* < 0.001 level.

**Table 6:** Correlating gender identity and gender expression.

Variable	Men	Masc	Enby	Andro	Women
Masc	0.385 **				
Enby	−0.767 **	−0.196 **			
Andro	−0.355 **	−0.137 **	0.443 **		
Women	−0.208 **	−0.232 **	−0.468 **	−0.185 **	
Fem	−0.292 **	−0.395 **	0.021 **	−0.041 **	0.371 **

** Correlation is significant at the 0.01 level (2-tailed).

**Table 7:** Predicting odds ratios for subgroups.

Predictor	OR	95% CI	*p*
Predicting low income (i.e., <25 k).			
fem(me) BIPOC	1.9238	0.482, 3.405	0.620
masc(uline) BIPOC	0.7549	0.197, 1.107	0.084
masc(uline) white	0.8049	0.548, 1.353	0.516
disabled white	1.0561	0.778, 2.105	0.331
disabled BIPOC	1.4035	0.806, 5.453	0.129
nondisabled BIPOC	0.9507	0.496, 4.175	0.504
Predicting feeling “very” or “extremely” safe in daily life.			
fem(me) BIPOC	0.7046	0.3410, 2.976	0.949
masc(uline) BIPOC	1.561	1.028, 6.205	0.043 *
masc(uline) white	1.1864	0.758, 1.889	0.442
disabled white	0.5505	0.233, 0.661	<0.001 ***
disabled BIPOC	0.7298	0.109, 0.728	0.009 **
nondisabled BIPOC	1.5701	0.196, 1.823	0.365

Note: The intersection between gender expression and race [represented by four categories: fem(me)BIPOC, masc(uline)BIPOC, fem(me)BIPOC, masc(uline)white] was analyzed using three dummy variables with fem(me) white respondents serving as the reference group. The intersection between ability and race (categorized into disabledwhite, disabledBIPOC, nondisabledwhite, nondisabledBIPOC) was represented by three dummy variables with nondisabled white respondents serving as the reference group for this analysis. * Significant at the *p* < 0.05 level. ** Significant at the *p* < 0.01 level. *** Significant at the *p* < 0.001 level.

**Table 8:** Predicting QOL by intersecting identities.

Predictor	b	SE	95% CI	t	*p*
(constant)	23.776	0.400	22.988, 24.565	59.380	<0.001 ***
femmeBIPOC	−0.096	0.700	−1.469, 1.277	−0.137	0.891
mascWHITE	0.428	0.339	−0.237, 1.094	1.262	0.207
mascBIPOC	0.622	0.704	−0.760, 2.004	0.884	0.377
disabledBIPOC	−1.149	0.664	−2.452, 0.153	−1.731	0.084
disabledWHITE	−1.378	0.370	−2.104, −0.653	−3.728	<0.001 ***
nondisabled BIPOC	0.791	0.800	−0.779, 2.362	0.989	0.323

Note: The intersection between gender expression and race [represented by four categories: fem(me)BIPOC, masc(uline)BIPOC, fem(me)BIPOC, masc(uline)white] was analyzed using three dummy variables with fem(me) white respondents serving as the reference group. The intersection between ability and race (categorized into disabledwhite, disabledBIPOC, nondisabledwhite, nondisabledBIPOC) was represented by three dummy variables with nondisabled white respondents serving as the reference group for this analysis. *** Significant at the *p* < 0.001 level.

**Table 9:** Predicting COVID-19 impact by intersecting identities.

Predictor	b	SE	95% CI	t	*p*
(constant)	3.465	0.094	3.280, 3.650	36.798	<0.001 ***
femmeBIPOC	0.129	0.191	−0.245, 0.503	0.676	0.499
mascWHITE	−0.210	0.094	−0.394, −0.026	−2.239	0.025 *
mascBIPOC	−0.154	0.168	−0.484, 0.175	−0.920	0.358
disabledBIPOC	−0.011	0.167	−0.338, 0.316	−0.064	0.949
disabledWHITE	0.093	0.101	−0.104, 0.291	0.928	0.354
nondisabled BIPOC	−0.399	0.204	−0.799, 0.001	−1.957	0.051

Note: The intersection between gender expression and race [represented by four categories: fem(me)BIPOC, masc(uline)BIPOC, fem(me)BIPOC, masc(uline)white] was analyzed using three dummy variables with fem(me) white respondents serving as the reference group. The intersection between ability and race (categorized into disabledwhite, disabledBIPOC, nondisabledwhite, nondisabledBIPOC) was represented by three dummy variables with nondisabled white respondents serving as the reference group for this analysis. * Significant at the *p* < 0.05 level. *** Significant at the *p* < 0.001 level.

**Table 10:** Vaccine preferences by demographics.

Variable		ASAP		Minimum Side Effects		After More Vulnerable Groups		Total	
		*n*	%	*n*	%	*n*	%	*n*	%
Age.									
	18–24	26	29.21%	33	37.08%	33	37.08%	89	19.82%
	25–34	93	40.43%	77	33.48%	79	34.35%	230	51.22%
	35–44	35	39.33%	25	28.09%	26	29.21%	89	19.82%
	45+	10	24.39%	15	36.59%	10	24.39%	41	9.13%
Gender.									
	women	18	35.29%	18	35.29%	12	23.53%	51	11.36%
	men	40	35.09%	43	37.72%	37	32.46%	114	25.39%
	nonbinary	106	37.32%	89	31.34%	99	34.86%	284	63.25%
Race.									
	White	127	37.91%	115	34.33%	119	35.52%	335	74.61%
	BIPOC	36	32.43%	34	30.63%	28	25.23%	111	24.72%
Disabled and/or Chronically Ill.									
	yes	116	39.19%	112	37.84%	103	34.80%	296	65.92%
	no	48	31.37%	38	24.84%	45	29.41%	153	34.08%
Income.									
	Under 25 k	66	32.04%	70	33.98%	72	34.95%	206	45.88%
	over 75 k	23	45.10%	14	27.45%	13	25.49%	51	11.36%
Live in the U.S.									
	yes	145	36.16%	137	34.16%	130	32.42%	401	89.31%
	no	19	39.58%	13	27.08%	18	37.50%	48	10.69%
Health conditions.									
	neither	14	12.28%	9	7.89%	10	8.77%	114	25.39%
	mental	84	44.92%	78	41.71%	85	45.45%	187	41.65%
	physical	3	27.27%	7	63.64%	4	36.36%	11	2.45%
	both	63	45.99%	56	40.88%	49	35.77%	137	30.51%
Total		164	36.53%	150	33.41%	148	32.96%	449	100%

## Data Availability

The data presented in this study are available on request from the corresponding author. The data are not publicly available due to ethical and privacy concerns.

## Appendix A

**Table A1:** Demographics and descriptive information.

		COVID Impact		COVID Care		COVID Work		Life Satisfaction		Health Self Report		Safe WHOQOL		Negative Feelings		Social Support		Total	
		Major or Catastrophic		7+ Days Outside of Home in Last Month		7+ Days Outside of Home in Last Month		Satisfied or Very Satisfied		Good to Excellent		Very Much or Extremely		Often/Always Past 2 Weeks		Usually/Always Receive Needed Support			
		*n*	%	*n*	%	*n*	%	*n*	%	*n*	%	*n*	%	*n*	%	*n*	%	*n*	%
Age																			
	18–24	38	42.70%	6	6.74%	34	38.20%	59	66.29%	54	60.67%	46	51.69%	47	52.81%	42	47.19%	89	19.82%
	25–34	109	47.39%	34	14.78%	67	29.13%	163	70.87%	149	64.78%	115	50.00%	134	58.26%	141	61.30%	230	51.22%
	35–44	39	43.82%	5	5.62%	30	33.71%	65	73.03%	61	68.54%	50	56.18%	62	69.66%	48	53.93%	89	19.82%
	45+	18	43.90%	8	19.51%	6	14.63%	25	60.98%	24	58.54%	23	56.10%	29	70.73%	17	41.46%	41	9.13%
Gender																			
	women	27	52.94%	3	5.88%	18	35.29%	32	62.75%	32	62.75%	23	45.10%	16	31.37%	25	49.02%	51	11.36%
	men	42	36.84%	16	14.04%	33	28.95%	83	72.81%	83	72.81%	69	60.53%	33	28.95%	61	53.51%	114	25.39%
	nonbinary	135	47.54%	36	12.68%	86	30.28%	197	69.37%	173	60.92%	141	49.65%	128	45.07%	162	57.04%	284	63.25%
Race																			
	white	154	45.97%	39	11.64%	100	29.85%	234	69.85%	221	65.97%	176	52.54%	132	39.40%	183	54.63%	335	74.61%
	BIPOC	48	43.24%	15	13.51%	37	33.33%	76	68.47%	66	59.46%	56	50.45%	43	38.74%	64	57.66%	111	24.72%
Disabled and/or Chronically Ill																			
	yes	146	49.32%	42	14.19%	91	30.74%	190	64.19%	157	53.04%	132	44.59%	133	44.93%	154	52.03%	296	65.92%
	no	57	37.25%	12	7.84%	46	30.07%	122	79.74%	131	85.62%	102	66.67%	44	28.76%	95	62.09%	153	34.08%
Income																			
	none	15	45.45%	4	12.12%	4	12.12%	20	60.61%	15	45.45%	16	48.48%	15	45.45%	18	54.55%	33	7.35%
	<20 k	62	46.27%	13	9.70%	42	31.34%	77	57.46%	77	57.46%	63	47.01%	58	43.28%	65	48.51%	134	29.84%
	20–40 k	68	53.13%	15	11.72%	49	38.28%	93	72.66%	78	60.94%	61	47.66%	52	40.63%	71	55.47%	128	28.51%
	40–75 k	39	37.86%	13	12.62%	28	27.18%	84	81.55%	79	76.70%	63	61.17%	38	36.89%	62	60.19%	103	22.94%
	75 k+	20	39.22%	9	17.65%	14	27.45%	38	74.51%	38	74.51%	30	58.82%	15	29.41%	33	64.71%	51	11.36%
Employed full time																			
	yes	74	37.56%	25	12.69%	79	40.10%	157	79.70%	146	74.11%	113	57.36%	70	35.53%	124	62.94%	197	43.88%
	no	130	51.59%	29	11.51%	58	23.02%	155	61.51%	142	56.35%	121	48.02%	107	42.46%	124	49.21%	252	56.12%
Live in the U.S.																			
	yes	186	46.38%	48	11.97%	126	31.42%	284	70.82%	256	63.84%	206	51.37%	164	40.90%	224	55.86%	401	89.31%
	no	18	37.50%	6	12.50%	11	22.92%	28	58.33%	32	66.67%	28	58.33%	13	27.08%	24	50.00%	48	10.69%
Total		204	45.43%	54	12.03%	137	30.51%	312	69.49%	288	64.14%	234	52.12%	177	39.42%	248	55.23%	449	100%
